# Gallium hydride vapor phase epitaxy of GaN nanowires

**DOI:** 10.1186/1556-276X-6-262

**Published:** 2011-03-28

**Authors:** Matthew Zervos, Andreas Othonos

**Affiliations:** 1 Nanostructured Materials and Devices Laboratory, Department of Mechanical Engineering, Materials Science Group, School of Engineering, University of Cyprus, P.O. Box 20537, Nicosia 1678, Cyprus; 2 Ultrafast Research Center, Department of Physics, University of Cyprus, P.O. Box 20537, Nicosia 1678, Cyprus

## Abstract

Straight GaN nanowires (NWs) with diameters of 50 nm, lengths up to 10 μm and a hexagonal wurtzite crystal structure have been grown at 900°C on 0.5 nm Au/Si(001) via the reaction of Ga with NH_3 _and N_2_:H_2_, where the H_2 _content was varied between 10 and 100%. The growth of high-quality GaN NWs depends critically on the thickness of Au and Ga vapor pressure while no deposition occurs on plain Si(001). Increasing the H_2 _content leads to an increase in the growth rate, a reduction in the areal density of the GaN NWs and a suppression of the underlying amorphous (α)-like GaN layer which occurs without H_2_. The increase in growth rate with H_2 _content is a direct consequence of the reaction of Ga with H_2 _which leads to the formation of Ga hydride that reacts efficiently with NH_3 _at the top of the GaN NWs. Moreover, the reduction in the areal density of the GaN NWs and suppression of the α-like GaN layer is attributed to the reaction of H_2 _with Ga in the immediate vicinity of the Au NPs. Finally, the incorporation of H_2 _leads to a significant improvement in the near band edge photoluminescence through a suppression of the non-radiative recombination via surface states which become passivated not only via H_2_, but also via a reduction of O_2_-related defects.

## Introduction

Group III-nitride (III-N) compound semiconductors such as GaN, InN, and AlN have been investigated intensively over the past decades in view of their successful application as electronic and optoelectronic devices [1]. In particular, III-N semiconductors are attractive since their band-gaps vary between 0.7 eV in InN [2] and 3.4 eV in GaN [3] up to 6.2 eV in AlN [4], allowing the band-gaps of Al*_x_*Ga_1-*x*_N or In*_x_*Ga_1-*x*_N to be tailored in between by varying *x *which is very important for the realization of high-efficiency, full spectrum solar cells. In addition III-N nanowires (NWs) have also been investigated in view of the up surging interest in nanoscale science and technology. More specifically, InN [5], GaN [6] NWs and also In*_x_*Ga_1-*x*_N NWs [7] have been grown and their transport and optical properties have been investigated. However, the use of III-N NWs for the fabrication of NWSCs has not yet been demonstrated. To date NWSCs have not only been fabricated from a single *p*-*i*-*n *core-shell Si NW [8], but also using disordered arrays of Si NWs [9]. Evidently the growth of high-quality GaN NWs is crucial for the fabrication of NWSCs based on III-N NWs. So far GaN NWs have not only been grown by a variety of methods including metal organic chemical vapor deposition (MOCVD), molecular beam epitaxy (MBE), but also via the direct nitridation of Ga with NH_3 _between 900 and 1100°C on a broad variety of substrates, e.g., SiC, Al_2_O_3_, and Si using various catalysts such as In, Fe, Ni, Au, and NiO, reviewed elsewhere [10]. The GaN NWs have a hexagonal-wurtzite crystal structure and their diameters vary between 10 and 50 nm. Nevertheless despite this broad variety of investigations there are still many issues pertaining to their growth and properties that need to be clarified and understood to improve crystal quality and to enable the fabrication of nanoscale devices such as NWSCs. Recently, hydride vapor phase epitaxy (HVPE) has been used to grow GaN layers [11] and also GaN NWs [12]. The use of H_2 _first of all eliminates O_2 _and secondly leads to the formation of Ga hydride, which in turn reacts with NH_3 _giving GaN. This method is cleaner compared to MOCVD or halide-VPE [13]. Previously, we showed that the use of a few % of H_2 _leads to the growth of straight GaN NWs with lengths of 2-3 μm and diameters of 50 nm [6,10]. More recently, Lim et al. [14] investigated the effect of H_2 _on the initial stages of growth of GaN NWs by varying the ratio of N_2_:H_2 _up to 0.6 and found that the density and growth rate of the GaN NWs decreased with increasing % H_2_. In this article, we have carried out a study into the growth of GaN NWs on Au/Si(001) via the reaction of Ga with NH_3 _and N_2_:H_2 _where the H_2 _content was varied between 10 and 100%. It has been find that the growth of straight GaN NWs on Au/Si(001) is critically dependent on the thickness of the Au and the Ga vapor pressure while no deposition occurs on plain Si(001). Increasing the H_2 _content leads to an increase in the growth rate, a reduction in the density of the GaN NWs and a clear suppression of the amorphous (α)-like GaN layer that forms without H_2_. A growth mechanism is proposed to explain these findings, where the effect of H_2 _is clarified in detail. Finally, we show that the incorporation of H_2 _leads to a significant improvement in the near band edge photoluminescence (PL) through a suppression of the non-radiative recombination via surface states and their passivation by H_2_.

## Experimental method

GaN NWs were grown using an atmospheric pressure CVD reactor described in detail elsewhere [10]. For the growth of GaN NWs, ≈0.1-0.5 g of Ga (Aldrich, Cyprus 99.99%) were used while square pieces of Si(001) ≈ 7 × 7 mm^2^, coated with 0.5 nm Au, were loaded only a few millimeters away from the Ga. The boat was always positioned directly above the thermocouple used to measure the heater temperature (*T*_H_) at the center of the 1" QT. After closing the reactor, 500 sccm of N_2_:10% H_2 _was introduced for 10 min. Then, the temperature was ramped to 900°C under a reduced flow of N_2_:(10-100%) H_2 _using a slow ramp rate of 10°C/min. Upon reaching 900°C, the same flow of N_2 _and H_2 _was maintained and 20 sccms of NH_3 _were introduced for 60 min after which the tube was allowed to cool down using the same gas flows during growth. The sample was removed only when the temperature was lower than 100°C. A summary of the relevant growth conditions is given in Table 1. The morphology of the GaN NWs was examined by a TESCAN scanning electron microscope (SEM) while their crystal structure and the phase purity were investigated by a SHIMADZU, XRD-6000 with a Cu-Ka source by performing a scan of θ-2θ in the range between 10° and 80°. Finally, PL measurements were carried out by exciting the GaN NWs at RT with λ = 290 nm.

**Table 1:** Summary of HVPE growth conditions for GaN NWs carried out on 0.5 nm Au/Si(001) at *T *= 900°C for 60 min via the reaction of Ga with 20 sccms of NH_3 _and N_2_:(10-100%) H_2_

	N_2 _(sccm)	H_2 _(sccm)	H_2 _(%)	*L *(μm)
CVD817	90	10	10	2.3
CVD818	40	10	20	3.4
CVD819	23	10	30	4.2
CVD821	15	10	40	4.7
CVD822	10	10	50	5.2
CVD823	-	100	100	11.3

Also listed are the average lengths of the GaN NWs.

## Results and discussion

**Figure 1:**
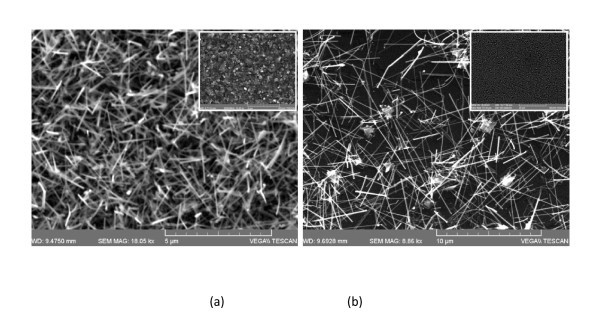
**SEM image of GaN NWs obtained using 10% H_2 _(a) and 100% H_2 _(b)** The inset in (a) shows the α-like GaN layer obtained with no H_2_, while the inset in (b) shows Au NPs obtained by heating 10 nm Au/Si(001) at 900°C using 100% H_2_. The Au NPs do not coalesce into larger clusters but remain isolated.

As described in detail elsewhere the direct reaction of Ga with NH_3 _using Ar as a carrier gas at 900°C leads to the growth of a few bent GaN NWs on top of an α-like GaN layer [10]. Such an α-like GaN layer, shown in the inset of Figure 1a, was obtained on 0.7 nm Au/Si(001) via the reaction of Ga and NH_3 _using Ar, under Ga-rich conditions at 10^-1 ^mBar. The α-like GaN layer is irregular and consists of connected crystallites that have sizes of ≈ 500 nm. It is important to point out that a low yield, non-uniform distribution of bent GaN NWs was obtained on top of this α-like GaN layer which was readily and clearly observed by SEM. On the contrary, no deposition took place on plain Si(001) in accordance with the findings of Hou and Hong [12] who found GaN NWs on patterned Au but not on plain Si in between the Au.

**Figure 2:**
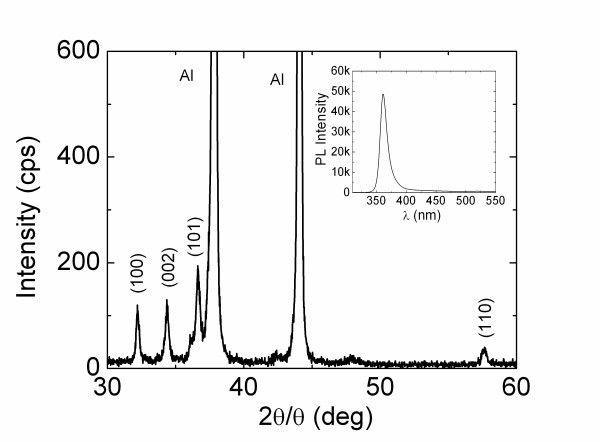
**XRD of the GaN NWs grown using 10% H_2 _with peaks corresponding to the (100), (002), (101) crystallographic planes of the hexagonal wurtzite structure of GaN**. The inset shows RT PL with a peak at 3.42 eV (≡362 nm).

GaN NWs were successfully grown on 0.7 nm Au/Si(001) via the direct reaction of Ga with NH_3 _at 900°C under a flow of 20 sccm NH_3 _and 90 sccm N_2_:10 sccm H_2_. The GaN NWs shown in Figure 1a had diameters of 50 nm and lengths up to 2 μm, confirming that Au does not inhibit their growth. More importantly, the GaN NWs are straight in agreement with the findings of Hou and Hong [12] who obtained long and bent GaN NWs using N and Ar and straight GaN NWs by adding only a few % H_2_. The GaN NWs grown using 10% H_2 _exhibited clear peaks in the XRD as shown in Figure 2 corresponding to GaN with a hexagonal wurtzite structure and lattice constants of *a *= 0.318 nm and *c *= 0.518 nm [10]. Excitation of the GaN NWs shown in Figure 1a using λ = 290 nm resulted in strong RT PL shown in the inset of Figure 2, where the prominent peak corresponds to band edge emission of GaN at 3.42 eV. Note that there was very little PL around 540 nm commonly referred to as the "yellow luminescence" band of GaN. Despite the improvement in the shape of the GaN NWs obtained with 10% H_2 _we found that the uniformity was poor over the Au/Si(001) surface due to the high boiling point of Ga, i.e., 1983°C and the resultant low vapor pressure at 900°C. The uniformity was improved significantly by fragmenting the Ga thereby increasing the vapor pressure, but this inadvertently led to the formation of connected crystallites or an α-like GaN layer.

The GaN NWs were not as straight as a direct consequence of the excessive Ga which is consistent with the morphology of the GaN NWs obtained under Ga-rich conditions by LPCVD [10]. A high yield, uniform distribution of straight GaN NWs over 1 cm^2 ^under these Ga-rich conditions was obtained by using 40% H_2 _while we observed a reduction in the areal density of the GaN NWs using 100% H_2 _and a significant enhancement in the growth rate.

This reduction in the areal density of the GaN NWs is consistent with the findings of Lim et al. [14] who observed a monotonic drop in the number of GaN NWs with increasing content of H_2 _which they attributed to the agglomeration of Au NPs. An alternative explanation for the observed reduction maybe the catalytic dissociation of H_2 _over the Au NPs which gives H that reacts with incoming Ga or Ga spreading out from the Au NPs to be explained in more detail below.

**Figure 3:**
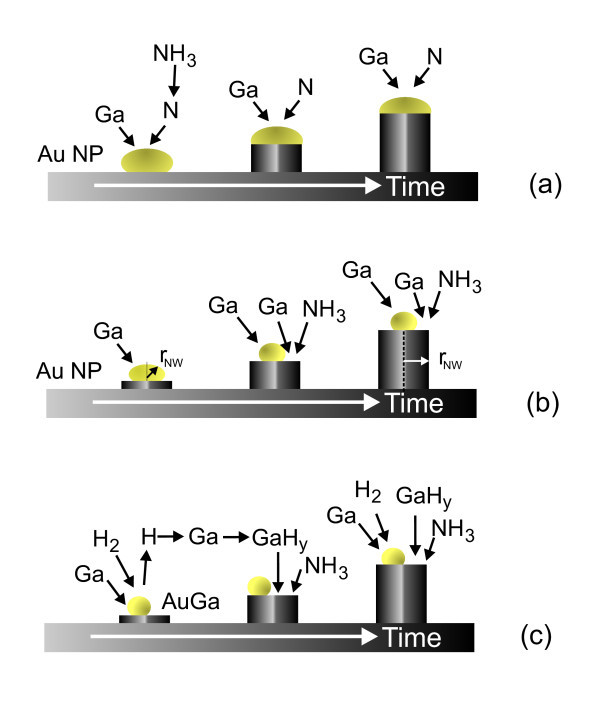
**Growth mechanisms of GaN NWs by VLS (a), self-regulated, diameter selective mechanism **[17]**(b), particle mediated, hydride-assisted growth via the catalytic dissociation of H_2 _at Au NPs (c)**.

In addition, we find that the growth rate becomes larger for 100% H_2_. The lengths of the GaN NWs grown under 100% H_2 _reached lengths >10 μm as shown in Figure 1b and Table 1. The growth rate is enhanced significantly because of a higher partial pressure of Ga hydride. Before we describe the growth mechanism which explains the reduction in the areal density of the GaN NWs, suppression of the α-like GaN layer, and higher growth rate, it is instructive to consider other growth mechanisms in more detail. The most commonly invoked mechanism on the growth of GaN NWs is the vapor-liquid-solid (VLS) mechanism whereby the Ga and N are suggested to enter the catalyst NP leading to the formation of GaN NWs as shown in Figure 3a. The poor yield of GaN NWs obtained with Au is usually attributed to the poor solubility of N in Au. Therefore, while Au is an efficient catalyst for the growth of other III-V NWs it has been suggested to be inactive in the case of GaN and Ni is commonly used as an alternative. Here, it should be pointed out that only a small fraction, i.e., ≈5% of NH_3 _molecules become thermally dissociated at 900°C; so, the availability of reactive N species is limited to begin with but the decomposition of NH_3 _over different metals is most effective in the following order: Ru > Ni > Rh > Co > Ir > Fe >> Pt > Cr > Pd > Cu >> Te [15]. Therefore, NH_3 _dissociates effectively over Ni but not Au, which makes Ni effective in the growth of GaN NWs. However, the formation energies of substitutional metal impurities, i.e., M = Au, Ni, on gallium sites (M_Ga_) and nitrogen sites (M_N_) have been calculated using *ab initio *pseudopotential electronic structure calculations and it has been found that Ni has a lower defect formation energy of 1.2 eV in GaN compared to 4 eV of Au [16]. In addition, Ni may oxidize in contrast to Au. Despite these limitations GaN NWs have been obtained using small Au NPs and a more careful analysis of the relation between the radii of the Au NP and GaN NW, carried out by Kuo et al. [17], led them to propose an alternative mechanism whereby the Ga enters the Au NP which sits on top of the GaN NW and forms a Au-Ga alloy but Ga also reacts with N at the top of the GaN NW outside and away from the Au NP as shown in Figure 3b. To be specific their GaN NWs had diameters, at least twice as large as the Au NPs and a self-regulated diameter selective growth model was put forward accounting for the stable growth of GaN NWs, where it was argued that the radius of the Au NP must be smaller than the radius of the GaN NW. This is in a way similar to the steady-state growth mechanism of GaN NWs by MBE whereby Ga atoms that impinge on the nanowire tip or within a surface diffusion length of the tip will incorporate. Adatoms arriving farther down the sides are likely to desorb rather than incorporate. Concerning GaN NWs, there is a general agreement concerning their steady-state growth regime but the nucleation process and the subsequent transient regime are, to some extent, a matter of controversy [18]. Interestingly, the distribution of GaN NWs we obtained with 100% H_2 _is very similar to that of Kuo et al. [17]. Now as seen above increasing the H_2 _content leads to a reduction in the areal density of the GaN NWs and the suppression of the α-like GaN layer. It is well known that noble metal NPs such as Au NPs are efficient in the catalytic dissociation of H_2 _and the formation of H which will react with incoming Ga around the Au NPs, leading to the formation of Ga hydride which is a gas [19,20]. It has also been shown that Ga species prefer to form Ga hydride in the temperature range 800-1000°C [21], so it is very likely that reactive Ga hydride will form at 900°C over the source of Ga but also in the vicinity of the Au NPs. One ought to recall that no GaN NWs grow on plain Si consistent with Hou and Hong [12], so Ga must enter the Au NPs and should spread out via alloying during the initial stages of growth [22]. The dissociation of H_2 _into H at the Au NP surface and the reaction of H_2_, H with incoming Ga or Ga spreading out from the Au NP will suppress the formation of the α-like GaN layer and the areal density of the GaN NWs.

At the same time, the Ga hydride released from the surface or generated upstream will promote one-dimensional growth via its reaction with NH_3 _at the tops of the GaN NWs as shown schematically in Figure 3c thereby enhancing the growth rate. The latter is essentially governed by the availability of reactive species at the tops of the GaN NWs in accordance with the self-regulated, diameter selective growth mechanism of Kuo et al. [17]. Finally, the reduction in the super saturation of the Au NPs will limit extreme fluctuations of the Ga in the Au NPs resulting in GaN NWs with uniform diameters and smooth surfaces. This in turn implies a reduction of surface states which are passivated by H_2 _giving stronger band edge PL emission.

## Conclusions

Straight GaN NWs with diameters of 50 nm, lengths up to 10 μm, and a hexagonal wurtzite crystal structure have been grown at 900°C on Au/Si(001) via the reaction of Ga with NH_3 _and N_2_:H_2 _where the H_2 _was varied between 10 and 100%. We find that the growth of high-quality GaN NWs can be achieved with Au having a thickness <1 nm. A growth mechanism was described whereby H_2 _reacts with Ga giving Ga hydride thereby promoting one-dimensional growth via its reaction with NH_3 _at the tops of the GaN NWs. Hydrogen may therefore be used not only to control the growth rate and obtain straight GaN NWs, but also to suppress the formation of the underlying α-like GaN under Ga-rich conditions.

## Abbreviations

HVPE: hydride vapor phase epitaxy; MBE: molecular beam epitaxy; MOCVD: metal organic chemical vapor deposition; NWs: nanowires; PL: photoluminescence; SEM: scanning electron microscope; VLS: vapor-liquid-solid.

## Competing interests

The authors declare that they have no competing interests.

## Authors' contributions

MZ carried out the growth, scanning electron microscopy and x-ray diffraction measurements. AO carried out the photoluminescence. All authors read and approved the final manuscript
